# Type I hypersensitivity promotes *Aedes aegypti* blood feeding

**DOI:** 10.1038/s41598-021-94416-w

**Published:** 2021-07-21

**Authors:** Michael J. Conway

**Affiliations:** Foundational Sciences, College of Medicine, Central Michigan University, Mount Pleasant, MI 48859 USA

**Keywords:** Immunology, Animal behaviour, Entomology

## Abstract

Mosquitoes play a major role in human disease by serving as vectors of pathogenic microorganisms. Mosquitoes inject saliva into host skin during the probing process. Mosquito saliva contains a number of proteins that facilitate blood feeding by preventing hemostasis. Mosquito saliva also contains potent allergens that induce type I hypersensitivity reactions in some individuals. Type I hypersensitivity reactions in skin involve IgE-mediated degranulation of mast cells, which leads to vasodilation and an itch sensation. We hypothesized that hypersensitivity to mosquito saliva influences blood feeding. To test this hypothesis, we recruited human subjects who consented to *Aedes aegypti* bites. We measured their first sensation of itch, the strength of their itch sensation, the number of times mosquitoes attempted to feed, the number of times mosquitoes probed their skin, feeding time, engorgement status, and wheal diameter. Here we show that hypersensitive subjects had a stronger itch sensation, and that the time to first itch sensation was inversely correlated with wheal diameter; however, mosquitoes tended to probe less and engorge more on these subjects. Follow-up experiments testing the impact of oral antihistamine treatment on mosquito feeding parameters failed to reveal a statistically significant result. Histamine also failed to promote blood feeding on an artificial membrane feeder. This study suggests that mosquito saliva-induced type I hypersensitivity promotes blood feeding but that this may be independent from histamine or histamine signaling.

## Introduction

Mosquitoes are the vectors of a number of human pathogens and transmit microorganisms while injecting saliva into host skin. Mosquito saliva contains a number of pharmacologically active proteins that serve to limit hemostasis largely by inducing vasodilation, inhibiting coagulation and platelet aggregation^1–3^. Mosquito saliva also contains a number of allergens that raise antibodies in exposed individuals, and some individuals develop type I hypersensitivity reactions to these allergens^4,5^.

Mosquito saliva allergens can induce both immediate type I hypersensitivity reactions and delayed type 4 hypersensitivity reactions. Immediate reactions likely occur as a result of anti-saliva allergen IgE antibodies binding to the surface of tissue-resident mast cells. Mast cells degranulate a number of effector molecules upon subsequent exposure to saliva allergen and cross-linking of IgE^6^.

Histamine is a major effector molecule that induces vasodilation and itch sensation^5^. Histamine alone is sufficient to induce the characteristic “wheal and flare” reaction that develops when sensitive individuals are exposed to mosquito saliva. The wheal is largely the result of vasodilation and fluid accumulation in the dermis. The flare is largely the result of chemical release from local nerve endings, causing vasodilation of surrounding cutaneous blood vessels and skin redness.

We hypothesized that the increased local fluid accumulation would promote mosquito blood feeding. To test this hypothesis, we recruited human subjects who consented to three to six *Aedes aegypti* bites. We measured their first sensation of itch, the strength of their itch sensation, feedings attempts, the number of times mosquitoes probed their skin, engorgement status, and wheal diameter. Subjects were classified as non-sensitive or hypersensitive based on the diameter of the wheal that developed after mosquito bite. We then determined the role of immune status on itch sensation, the number of times mosquitoes probed their skin, and engorgement status.

We found that hypersensitive subjects developed a stronger itch sensation, and that the diameter of the resulting wheal inversely correlated with the first sensation of itch. Hypersensitive subjects also tended to experience fewer probing attempts, which correlated with a significant increase in blood engorgement. However, oral antihistamine treatment failed to significantly influence mosquito feeding. Histamine treatment in an artificial blood meal also did not promote blood feeding.

This study identifies mosquito saliva-induced type I hypersensitivity as an evolutionary strategy used to increase blood feeding. Reducing the number of probing attempts and increasing the volume of blood obtained during engorgement increases the likelihood that a mosquito acquires sufficient nutrition to support oogenesis. Oral antihistamine and exogenous histamine treatments failed to influence mosquito feeding, which suggests that type I hypersensitivity promotes blood feeding in a way that is independent from histamine or histamine signaling.

## Methods

### Ethics statement

The Institutional Review Board at Central Michigan University approved human subject research described in this manuscript (Protocol #1083224-3 and #1209923-2). All methods were performed in accordance with relevant guidelines and regulations. Participants were 18 years of age or older, they were not currently on any medications, and had no history of severe allergic reactions. Written informed consent was obtained from each participant.

### Mosquito rearing

*Aedes aegypti* (Rockefeller strain) were maintained on a sugar solution at 27 °C and 80% humidity according to standard rearing procedures. Mosquitoes were fed defibrinated sheep’s blood (Hemostat) using a Hemotek artificial membrane feeder with and without 100 nmol/L histamine (Sigma).

### Itch sensation and mosquito feeding parameters

One 1–2-week-old female *A. aegypti* was placed in a small plastic cupule with metal screen mesh and placed on the left forearm for blood feeding. Subjects reported when they felt the first sensation of itch and the strength of the itch on visual analogue scales^7^. The first sensation of itch was reported on a 0–5 min scale. The strength of the itch was reported on a 0–10 scale where 10 represented an itch sensation so strong that they would have to remove the mosquito from their arm. We asked subjects to report their maximum itch sensation on visual analogue scales during the 5 min feeding session. The number of feeding attempts were defined as any mosquito that initiated probing within 5 min. Additionally, the number of times the mosquito probed the subject’s skin was assessed, in addition to its engorgement status on a scale of 0–5^8^, the length of time it fed on the subject, and the diameter of the resulting wheal. Individuals with wheals greater or equal to 5 mm in diameter were considered hypersensitive, which is consistent with previous literature^9^. Three mosquitoes attempted to feed on each subject sequentially. For the oral antihistamine experiment, subjects were enrolled and were subjected to up to 6 mosquito bites. The same itch and feeding parameters were measured as stated above. At least one week later, the experiment was repeated after oral administration of 10 mg cetirizine-HCl (Zyrtec). Mosquitoes did not feed on subjects until 1 h after oral antihistamine treatment.

### Statistical analysis

**Figure 1:**
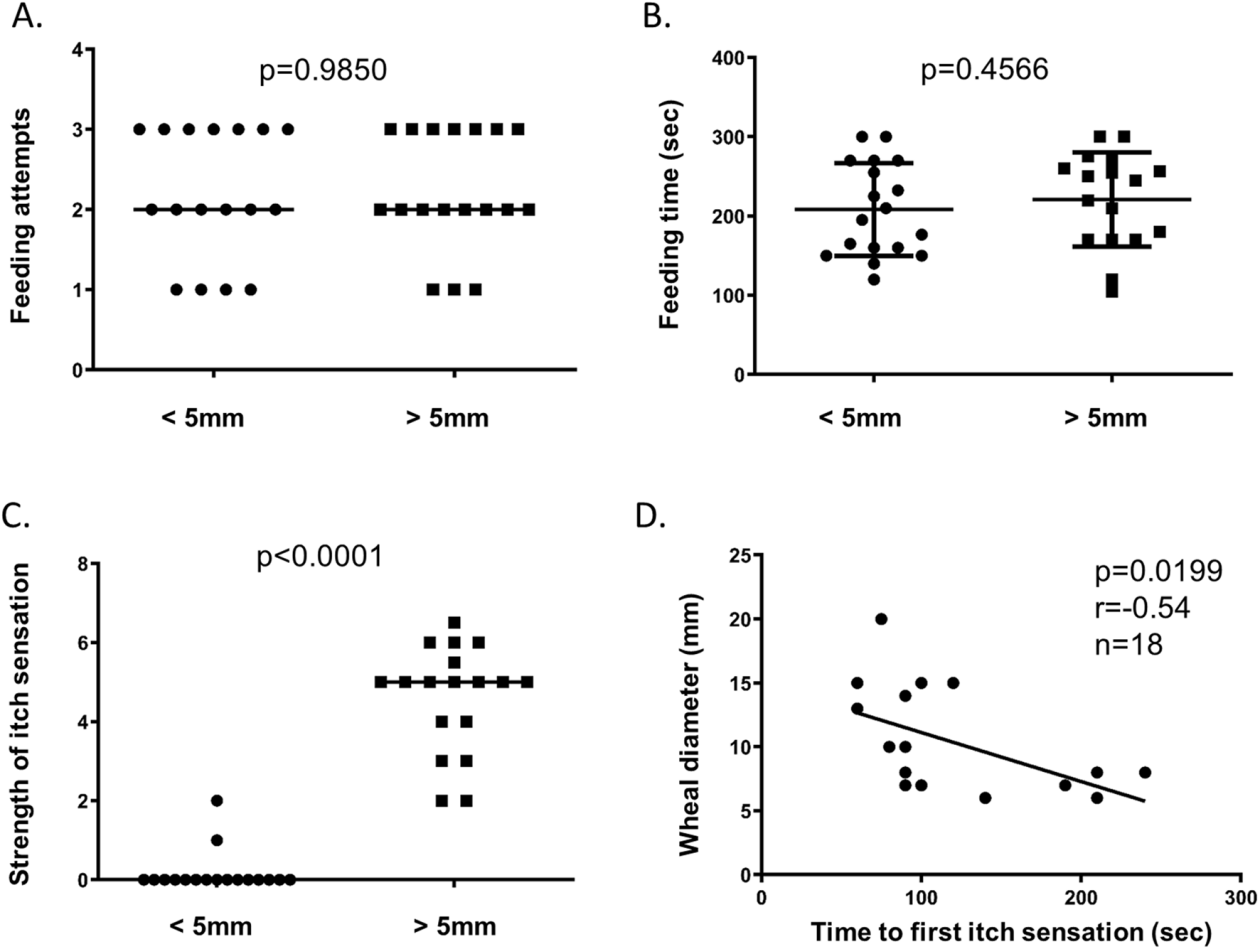
Mosquito saliva-induced type I hypersensitivity induces strong itch sensation in humans. Three mosquitoes were allowed to feed on the left forearms of non-sensitive (< 5 mm) and hypersensitive (> 5 mm) human participants. (**A**) The number of mosquitoes that attempted to feed per human participant were quantified. (**B**) Mosquitoes were given five minutes to feed on human participants once probing initiated and the feeding time was quantified in seconds. The average feeding times per human participant are shown. (**C**) The strength of the itch sensation experienced by non-sensitive and hypersensitive human participants was rated on a 0–10 scale. (**D**) Correlation analysis was performed between the wheal diameter and the time to first itch sensation in hypersensitive participants. Significance (*p*), Pearson’s coefficient (*r*), and total number of hypersensitive individuals (n) are shown. Statistical significance between groups was determined after testing for normal distribution using the Kolmogorov–Smirnov test. Student’s t tests were used for normally distributed data. Mann–Whitney tests were used for data that was not normally distributed.

Kolmogorov–Smirnov normality tests were performed on all datasets prior to assigning specific statistical tests. Data that passed a normality test were in Figs. 1B, 3A,B,E and 4. Student’s t tests were used to assess statistical significance in datasets that passed a normality test. Remaining datasets that failed the normality test were assessed using Mann–Whitney tests. Data is presented and statistical tests were performed using averaged data that was compiled from individual human subjects.

## Results

### Mosquito saliva-induced type I hypersensitivity induces a strong itch sensation in human subjects

Mosquito saliva allergens can induce type I hypersensitivity reactions in some individuals, which occurs as a result of vasodilation and inflammation^5^. We hypothesized that type I hypersensitivity reactions influence mosquito blood feeding. To test this hypothesis, we enrolled human subjects to participate in a mosquito feeding study. Participants were at least 18 years of age, they were not currently on any medication, and they had no history of severe allergic reaction. Thirty-five human subjects were enrolled and completed the study. Fifty-seven percent of subjects were male and 43 percent were female. The median age was 20, the average age was 26, and there was a range of 18–52 years old (Table 1).

**Table 1:** Subject characteristics.

Identifier	Sex	Age	Wheal diameter^a^	Hypersensitive^b^	First itch sensation (sec)
1	Male	36	15	Yes	60
2	Male	18	4	No	n/a
3	Female	36	4	No	n/a
4	Female	20	3	No	90
5	Male	19	15	Yes	100
6	Female	20	4	No	n/a
7	Male	20	6	Yes	135
8	Male	26	3	No	n/a
9	Male	18	3	No	n/a
10	Male	25	8	Yes	240
11	Female	19	8	Yes	210
12	Female	40	3	No	n/a
13	Female	52	0	No	n/a
14	Male	37	13	Yes	60
15	Female	18	7	Yes	200
16	Female	18	3	No	n/a
17	Male	48	3	No	n/a
18	Female	18	4	No	n/a
19	Male	18	7	Yes	190
20	Female	52	3	No	n/a
21	Female	20	14	Yes	90
22	Female	20	8	Yes	90
23	Male	27	3	No	n/a
24	Female	24	3	No	n/a/
25	Male	27	4	No	n/a
26	Male	19	8	Yes	90
27	Female	20	6	Yes	210
28	Female	18	2	No	n/a
29	Male	36	10	Yes	80
30	Male	20	7	Yes	70
31	Male	21	10	Yes	90
32	Male	19	7	Yes	100
33	Male	24	4	No	60
34	Male	21	15	Yes	120
35	Male	37	20	Yes	75

^a^Largest wheal diameter per subject in mm.

^b^Hypersensitive was defined as any subject with a wheal > 5 mm in diameter.

In order to facilitate mosquito feeding, a 1–2-week-old female *Aedes aegypti* was placed in a small plastic cupule covered by a metal mesh screen. Each human subject was exposed to three mosquitoes on their left forearm and mosquitoes were allowed an opportunity to feed. A feeding attempt was defined as any mosquito that initiated probing within 5 min. Subjects were categorized as non-sensitive if their wheals were less than 5 mm and hypersensitive if they had a wheal that was more than 5 mm. The same number of feeding attempts were observed for both non-sensitive and hypersensitive subjects (Fig. 1A). Mosquitoes also took the same amount of time to feed on both non-sensitive and hypersensitive subjects (Fig. 1B). Mosquitoes were given 5 min to feed once probing initiated, which was enough time for feeding mosquitoes to become fully engorged.

We also determined the time when subjects reported their first itch sensation, which ranged from 60 to 240 s. Hypersensitive subjects were more likely to report an itch sensation (Table 1). Both non-sensitive and hypersensitive subjects also rated the strength of their itch sensation on a 0–10 visual analogue scale where 10 was described as an itch sensation so strong that mosquito feeding would have to be interrupted^10^. There was a significant difference between the strength of itch sensation experienced by non-sensitive and hypersensitive individuals (Fig. 1C). Additionally, the diameter of the wheal that developed in hypersensitive individuals was inversely correlated to the time to first itch sensation (Fig. 1D). These data suggest that *A. aegypti* attempt to feed on non-sensitive and hypersensitive individuals similarly, but that hypersensitive individuals have a significant itch sensation that is sensed early when there is a robust wheal and flare reaction.

### Mosquito saliva-induced type I hypersensitivity promotes blood feeding in human subjects

**Figure 2:**
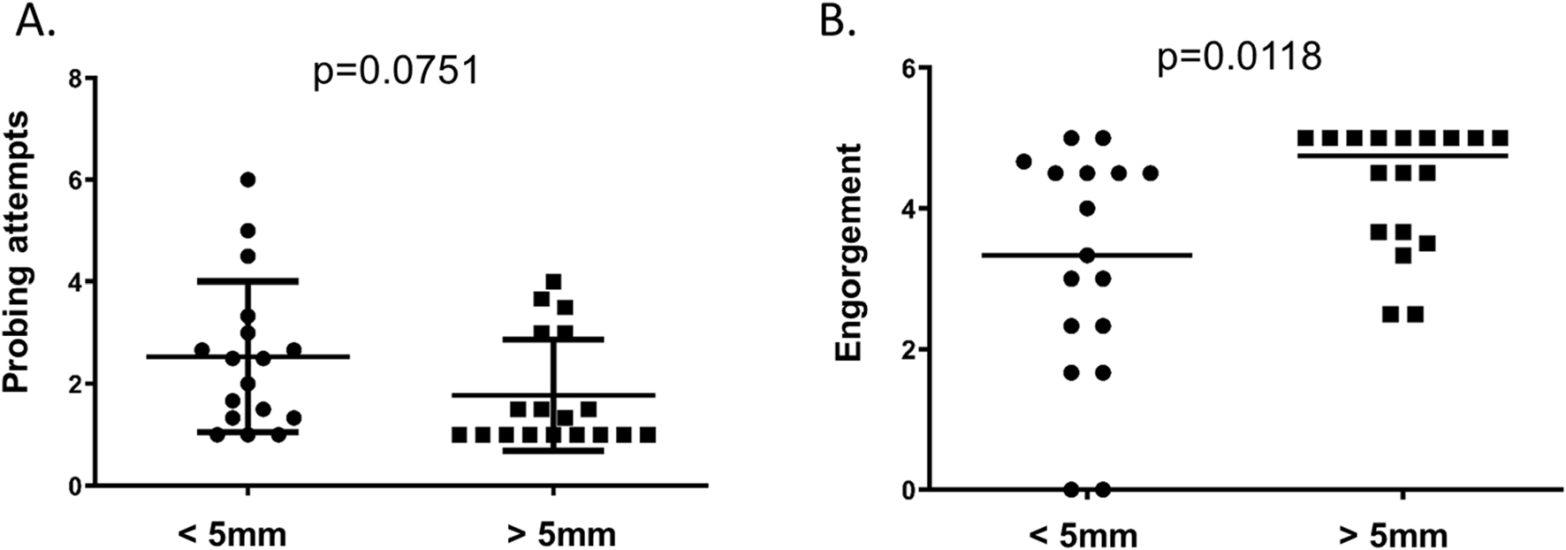
Mosquitoes feed better on hypersensitive subjects. Up to three mosquitoes probed the left forearms of non-sensitive (< 5 mm) and hypersensitive (> 5 mm) human subjects for five minutes. (**A**) The average number of probing attempts was quantified per human subject. (**B**) Mosquito engorgement was quantified on a 0–5 scale. Statistical significance between groups was determined after testing for normal distribution using the Kolmogorov–Smirnov test. Student’s t tests were used for normally distributed data. Mann–Whitney tests were used for data that was not normally distributed.

In order to determine if type I hypersensitivity influences mosquito blood feeding, mosquitoes that attempted to feed were monitored for the number of times they probed a subject’s skin, and their resulting engorgement status was rated on a 0–5 scale^8^. We compared the average of mosquito probing attempts on individual human subjects (Fig. 2A). This showed a trend toward fewer probing attempts on hypersensitive subjects, but the result was not statistically significant. Interestingly, more mosquitoes failed to fully engorge on non-sensitive individuals (Fig. 2B).

### Influence of oral antihistamine treatment on mosquito feeding

We hypothesized that histamine released during type I hypersensitivity promotes blood feeding. In order to directly test the role of histamine in mosquito blood feeding, we enrolled non-sensitive and hypersensitive human subjects who consented to mosquito bites after treatment with an over-the-counter oral antihistamine (10 mg cetirizine-HCl) (Table 2). Cetirizine is a potent second-generation antihistamine that is a selective antagonist of the histamine H_1_ receptor. The histamine H_1_ receptor is expressed in smooth muscles and vascular endothelial cells and is activated by biogenic amine histamine that is released during mast cell degranulation. Previous literature supports that 10 mg cetirizine-HCl effectively reduces the development of wheal and flare during mosquito bite challenge and therefore inhibits histamine’s role in type I hypersensitivity^9,11^.

**Table 2:** Subject characteristics from antihistamine study.

Identifier	Sex	Age	Wheal diameter^a^	Hypersensitive^b^
1	Female	18	0	No
2	Male	37	20	Yes
3	Male	20	7	Yes
4	Male	21	10	Yes
5	Male	19	7	Yes
6	Male	27	4	No
7	Female	24	3	No
8	Male	27	3	No
9	Male	24	4	No
10	Female	20	6	Yes

^a^Largest wheal diameter per subject in mm.

^b^Hypersensitive was defined as any subject with a wheal > 5 mm in diameter.

**Figure 3:**
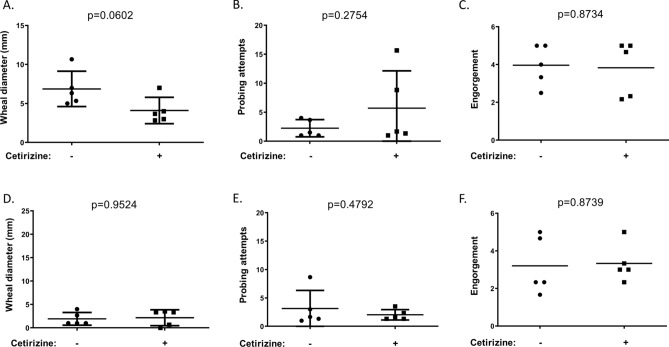
Influence of oral antihistamine treatment on mosquito feeding. A total of six mosquitoes were allowed to feed on the forearms of (**A**–**C**) hypersensitive or (**D**–**F**) non-sensitive human subjects without (−) and with ( +) oral antihistamine treatment. (**A**,**D**) The wheal diameter that developed after each mosquito bite was quantified. (**B**,**E**) The number of probing attempts was quantified. (**C**,**F**) Mosquito engorgement was quantified on a 0–5 scale. Statistical significance between groups was determined after testing for normal distribution using the Kolmogorov–Smirnov test. Student’s t tests were used for normally distributed data. Mann–Whitney tests were used for data that was not normally distributed.

**Figure 4:**
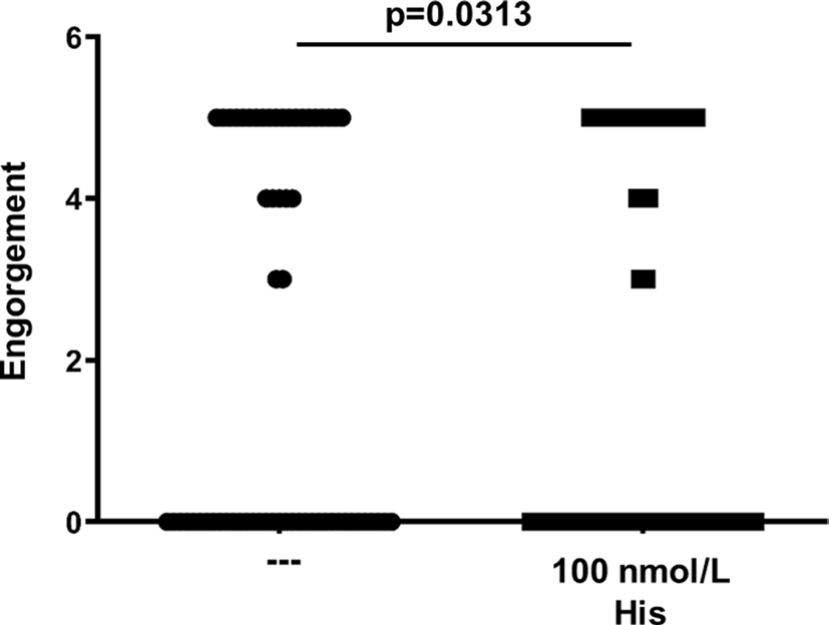
Exogenous histamine does not impact engorgement when blood is provided as an artificial bloodmeal. Three groups of 20 *A. aegypti* were fed either normal defibrinated sheep’s blood (--) or defibrinated sheep’s blood with 100 nmol/L histamine using a Hemotek artificial membrane feeder for 30 min. Mosquito engorgement was quantified on a 0–5 scale. Statistical significance between groups was determined after testing for normal distribution using the Kolmogorov–Smirnov test. Student’s t tests were used for normally distributed data. Mann–Whitney tests were used for data that was not normally distributed.

Human subjects allowed up to six mosquitoes to feed on their left and right forearms (three bites per arm). The wheal diameter, number of probing attempts, and engorgement status were determined. This experiment was performed without oral antihistamine treatment and at least one week later with oral antihistamine treatment. Mosquitoes were allowed to feed 1 h after treatment with 10 mg cetirizine-HCl. Cetirizine-HCl treatment tended to decrease wheal diameter, although the reduction was not statistically significant likely due to the low number of participants (Fig. 3A). There was also a trend leading to an increase in probing attempts, which was not statistically significant (Fig. 3B). There was no difference in engorgement between groups (Fig. 3C). Cetirizine-HCl had no impact on wheal diameter, probing attempts, or engorgement in non-sensitive individuals (Fig. 3D–F). We also tested if exogenous histamine promotes blood feeding alone by feeding three groups of 20 *A. aegypti* either defibrinated sheep’s blood or defibrinated sheep’s blood containing 100 nmol/L histamine using a Hemotek membrane feeding system^12,13^. The presence of histamine appeared to slightly decrease mosquito feeding (Fig. 4).

## Discussion

*Aedes aegypti* salivary glands express over one hundred secreted proteins that have been classified as D7 proteins, phosphatidylethanolamine binding proteins, odorant and juvenile hormone binding proteins, serpins and other protease inhibitors, a sialokinin vasodilator, nucleotidases, serine proteases, sugar digestion related proteins and other enzymes, lectins, angiopoietins, anti-microbial proteins and peptides, mucins and peritrophins, antigen 5 proteins, and many more proteins of unknown function^3,14–17^. Saliva of all hematophagous arthropods appear to have anti-coagulant, anti-platelet, and vasodilatory activities, which reduce hemostasis and promote blood feeding. Mosquito saliva also contains proteins that are allergens, and they can induce both immediate type I hypersensitivity reactions and delayed type 4 hypersensitivity reaction in sensitized individuals.

*Aedes aegypti* has three major saliva allergens named Aed a 1–3^5^. Immediate reactions are likely formed after engagement of these allergens with IgE-bound mast cells. Cross-linking of IgE and saliva allergens leads to degranulation of a number of effector molecules including pro-inflammatory cytokines and histamine, which induces vasodilation and an itch sensation. Histamine-induced vasodilation is sufficient to form the classic “wheal and flare” reaction that is characterized by an influx of fluid into the dermis^11,18^.

It is theoretically possible that histamine-induced vasodilation contributes to reducing hemostasis by increasing fluid and pressure in the feeding site and promotes blood feeding in mosquitoes. Previous research revealed that delayed type hypersensitivity to *Phlebotomus papatasi* saliva allergens promotes sand fly feeding in an animal model and in humans^19^. This was likely due to increased blood flow at feeding sites, which was measured by the laser Doppler method^19^. Previous research has also shown that histamine delivered by skin prick can increase blood flow as measured by the laser Doppler method. Increased blood flow was detectable in wheal and flare lesions and occurred within 2 min of exposure^18^.

We tested our hypothesis that mosquito saliva-induced hypersensitivity promotes blood feeding by allowing *A. aegypti* to feed on human subjects and then determined the subject’s first itch sensation, the strength of their itch sensation, the wheal diameter, and a number of mosquito feeding parameters including the number of feeding attempts, the number of times mosquitoes probed their skin, and engorgement status. It’s important to note that itch sensation is a subjective experience, although itch sensation is commonly quantified using visual analogue and numerical rating scales similar to assessment of pain^7,10^. Some studies that quantify itch sensation suffer because of delayed reporting, which can negatively influence the individual’s memory of their itch sensation.

We asked subjects to report their maximum itch sensation on visual analogue scales during their feeding session. This strategy avoided any issues related to a delay in reporting. We found that only hypersensitive subjects experienced a strong itch sensation. Only two non-sensitive individuals experienced mild itch sensation. It is likely that these non-sensitive individuals sensed the probing process rather than itch stimulated by hypersensitivity.

We also found that the time to first itch sensation inversely correlated with wheal diameter—larger wheals resulted in earlier detection and smaller wheals resulted in later detection. Although all hypersensitive subjects experienced an itch sensation, mosquitoes were more likely to fully engorge on these subjects. Importantly, we excluded the possibility that hypersensitive subjects were simply easier to feed on by quantifying the number of feeding attempts in non-sensitive and hypersensitive subjects. There was no difference in feeding attempts, which suggests that mosquitoes do not discriminate between individuals based on hypersensitivity status. The number of probing attempts trended higher in non-sensitive individuals and engorgement was reduced in these individuals.

We hypothesized that type I hypersensitivity promoted blood feeding and increased the likelihood that a mosquito would fully engorge. Accordingly, we tested if oral antihistamine treatment could reduce mosquito bite reactions and modify feeding parameters. Oral antihistamine appeared to reduce wheal diameter, although the limited number of study participants likely prevented us from achieving statistical significance. There was no statistically significant difference in probing attempts or engorgement in hypersensitive subjects treated with oral antihistamine. Oral antihistamine treatment also had no effect on non-sensitive subjects.

These data suggest that mosquito saliva allergens contribute to feeding success but that this process may be independent from histamine or histamine signaling. A more complex picture emerges when previous literature is assessed. For instance, one of the most abundant proteins in mosquito saliva are D7 proteins. D7 proteins bind to biogenic amines like histamine, which may reduce itch sensation^1,20^. It’s important to note that, despite the abundance of D7 proteins in mosquito saliva, itch sensation is experienced by many individuals who are bitten by mosquitoes. This suggests that D7 proteins are unable to completely inhibit type I hypersensitivity. It is possible that in nature a mild hypersensitivity reaction promotes blood feeding without inducing an early itch response. A strong hypersensitivity reaction can promote blood feeding under controlled conditions, but the induction of a strong and early itch response would likely lead to rapid detection and removal of the biting insect. In support of this hypothesis, our study did not reveal any added benefit to strong hypersensitivity reactions, and instead found that large wheal and flare reactions lead to earlier itch sensations.

Other studies suggest that proteases released during mast cell degranulation may target arthropod saliva proteins and inactivate them^21^. It is unclear how proteolytic degradation of mosquito saliva proteins would impact feeding, but our data suggests that hypersensitivity reactions benefit feeding under controlled conditions. Future research is needed to determine the parameters of type I hypersensitivity that benefit mosquito feeding and if there is a “sweet spot” that maximizes blood feeding and avoiding detection. We also need to determine if mosquito saliva allergen epitopes are under positive selection, and if they have evolved to induce hypersensitivity and promote blood feeding. Further research needs to occur to exclude the role of histamine and histamine signaling in mosquito feeding, and it will be important to determine if hypersensitivity plays a role in the epidemiology of vector-borne diseases.

## Supplementary Information

Supplementary Information 1.

Supplementary Information 2.

Supplementary Information 3.
